# Aggression, Erection, and Masturbation in Feral Pottoka Ponies and Implications for Equine Welfare

**DOI:** 10.3390/ani12040421

**Published:** 2022-02-10

**Authors:** Katherine Grillaert

**Affiliations:** The Family Horse, Wales, WI 53183, USA; katie@thefamilyhorse.com

**Keywords:** equine, welfare, behavior, training, unwanted, stallion, gelding, management, horse–human, interactions

## Abstract

**Simple Summary:**

Horse trainers and handlers have expressed concerns that equine erection and masturbation may be associated with unwanted or dangerous behaviors. Such concerns and attitudes may guide decisions that affect the welfare of male horses. In this study, male feral ponies were observed before, during, and after natural occurrences of erection and masturbation. Erection and masturbation were not associated with reproductive or aggressive behavior, and they did not predict a change in intensity or energy of behavior. Rather, they were often performed in relaxed moments, with the pony displaying calm behaviors. Understanding the natural presentation of these behaviors can help handlers and trainers to interpret them when in the context of horse-human interactions.

**Abstract:**

Erection and masturbation in horses are considered unwanted behaviors in training contexts, despite recognition that these are naturally occurring behaviors that are integral to the welfare of male horses. Equestrians, especially those who use positive reinforcement in their training, expressed concern that the presence of such behaviors might be associated with aggressive or sexual behaviors aimed at humans participating in horse-human interactions. The implications of such attitudes could negatively affect male horses by excluding them from welfare-friendly training systems. In this study, feral stallions were observed to describe and quantify behaviors that occurred before, during, and after erection and masturbation, and to identify change in arousal. This study did not find evidence that erection and masturbation are associated with increases in arousal, or to sexual or aggressive behavior toward other horses. The possible presence or induction of erection or masturbation alone might not limit male horses from participating in certain handling, training, or riding contexts. These findings, along with further research, may be used to inform interpretations of horse–human interactions that involve erection or masturbation.

## 1. Introduction

Spontaneous erection and masturbation (SEAM) in male horses were considered undesirable behaviors in breeding, handling, and training contexts. This term is used to define penile tumescence (erection) and bouncing, pressing, or sliding of the penis against the belly (masturbation) when the horse is alone or with other male horses, (i.e., not immediately in a breeding situation with a female horse) [1]. In mammals, erection and masturbation are achieved through elaborate interactions between the central nervous system, the vascular and smooth muscle systems, hormones, and psychological and environmental factors [2,3], though with species-specific differences [4]. Spontaneous erection (occurring outside of a reproductive context) appears common in many species, including donkeys [5], gayal [6], sheep [7], deers [8], cats [9], dolphins [10], elephants [11], rhinoceroses [12], monkeys [13], Sprague–Dawley rats [14], dogs [15], and human males [16]. Human attitudes toward SEAM in stallions are not always favorable [17], and the consequences of these attitudes included the use of antierection and antimasturbatory devices such as stallion “brushes”, “rings”, and “cages” [18], electronic shock devices [17], as well as vocal and physical corrections with whips, lead ropes, forced trotting, and other punishments to terminate and suppress the behavior [19,20,21].

A series of studies of stallion fertility and SEAM by McDonnell and colleagues have refuted the hypothesis that the behavior interfered with breeding performance [19]. Stallions did not engage in SEAM to the detriment of libido or performance [19]. Further, aversive conditioning of SEAM leads to suppressed breeding behavior and reduced semen quality [19]. These findings led to recommendations for equine breeders to eliminate the use of aversive anti-SEAM devices and conditioning [19].

There is a growing focus on understanding the opportunity to perform SEAM from a welfare perspective. Rather than being an abnormal behavior performed due to social isolation or sexual frustration [22,23], SEAM occurs regularly in stabled, pastured, and group-housed geldings and stallions, as well as in stallions with daily breeding opportunities [1]. It was also observed in several populations of zoo and feral horses [22,24,25,26]. In addition to being performed in a variety of environments, both natural and human-influenced, there seems to be remarkable similarity in the duration and frequency of SEAM between stabled horse and pony stallions and pastured horse, pony, and donkey stallions [1], suggesting a highly conserved genetic basis for the behavior [27] (although a study of seven stabled stallions by Tischner et al. reported substantially fewer episodes of masturbation per day than the McDonnell studies [23]). SEAM is also observed with regularity in horse and pony geldings, although at lower frequencies and decreased duration [1]. Such “naturalness” is argued to promote biological functioning and is further considered an ethological need if it contributes to a positive experiential state. Therefore, it should be considered when assessing an animal’s welfare [28]. Furthermore, as SEAM is a naturally occurring behavior in feral horses, it should also not be considered a stable vice or stereotypical behavior [1,29,30]. This can be contrasted for example with wind-sucking or weaving, behaviors that are only observed in domestic horses and considered a “disease of domestication” [31]. Accordingly, the animal should have regular opportunities to express SEAM, and such behaviors should not be punished wholesale.

Despite advances in understanding SEAM, it remains a controversial behavior when present during training, riding, and handling, and is of particular discussion at conferences, on social media, and in the blogs of equestrians who espouse the use of positive reinforcement applications [20,32,33]. Punctuating such discourse are concerns of the display or development of aggressive or sexual behavior of the male horse toward the human if SEAM [20] is present, and the possible correlation of SEAM with other behaviors (e.g., pushiness, frustration, over-arousal) that may interfere with training [33]. While equine researchers are beginning to study the association between clicker training and SEAM [32], results were not yet published. Meanwhile, the worry that the use of appetitive techniques encourages unwanted behavior in stallions and geldings may lead to them being excluded from training systems that emphasize positive reinforcement. These systems decrease fear, improve motivation to learn, reduce stress, and reduce problem behavior, i.e., animals trained in this system have improved welfare compared to that of those trained in (usually traditional) aversive methods [34,35,36,37,38,39], though see Lesimple [40] regarding the interpretation of equine welfare indicators. Thus, to indiscriminately exclude stallions would be at odds with the positive duty for equine caretakers to “provide positive mental experiences” [41]. Rather, there is a need for further research regarding the physiological and mental experiences of horses that display erection or masturbation (E/M) during training and handling contexts.

Understanding natural behavior is necessary to evaluate the behavior and welfare of domestic horses that have close horse-human interactions [42], and this is no different when considering SEAM. While previous studies described and quantified SEAM, there is little published information on the behaviors occurring before, concurrent with, and after the presentation of SEAM. In particular, ethological observations of these behaviors are crucial to considering and designing appropriate training and management systems that both allow the male horse to express normal behavior and scaffold successful human–horse interactions [43,44].

A previous study by McDonnell and colleagues identified behaviors that occurred before, during, and after SEAM in stalled horses (observed two minutes before and two minutes after, behaviors observed were feeding, resting standing, sternal or lateral recumbency, drinking, walking, autogrooming), finding no statistical difference in these “endpoint” behaviors between stallions in box stalls and those in tie stalls [1].

The present study differs in a few important ways. First, this study reports the relative frequency and type of observed behaviors, whereas these data were not published in the McDonnell studies. Furthermore, the subjects in this study were feral, allowing for the expression and observation of the full repertoire of social and individual (nonsocial) behavior. Importantly, this includes aggressive and sexual behavior, both of which are of concern to equestrians. Therefore, this study design allows for ethological exploration of the behaviors preceding, during, and after SEAM. It also investigates the occurrence of behaviors of concern in equine handling and training. Finally, a thorough understanding of horse–horse interactions in natural environments critically informs the framing of analogous human–horse social interactions. If the welfare of stallions and geldings is to be considered seriously, scientists and practitioners must take into account the natural expression and occurrence of SEAM and the resulting implications for domestic horse handling and training.

## 2. Materials and Methods

### 2.1. Subjects

Pottoka, or Basque Ponies (*Equus ferus caballus*), are an endangered, semiferal breed endemic to the Basque Country of Spain and France [45]. Observations were conducted of privately owned Pottoka ponies living in the Gredos mountains in Northern Extremadura, Spain. The ponies are free-roaming across 1000 hectares, where they are unhandled and unmanaged, except for periodic culls designed to maintain social stability and natural male:female ratio.

The subjects were eight male ponies between the ages of 1–10 years, across four distinct bands (see Table 1). Three bands had one mature harem stallion each, and one pony was a yearling in his sire’s band. These bands consisted of mares and their yearlings and foal offspring. No male ponies were gelded. The fourth band was a bachelor band consisting of four young colts aged 2–4 years.

The study was conducted from 17–22 May 2015, which coincided with foaling and subsequent breeding when females returned to estrus after foaling. Observations were made between 8 a.m. and 8 p.m., dependent upon locating the ponies in the mountains. During the study, the weather was sunny or overcast, with no precipitation. The daytime temperature ranged from 15–33 C.

### 2.2. Observation of Behavior

Focal animal sampling (male ponies in harems) and all animal sampling (all ponies in the bachelor band) methods were used with all occurrences sampling to observe and record each incidence of E/M in male ponies. Behavior that occurred within 60 s before, concurrently, and within 60 s after bouts of E/M was recorded. Using this method, all male animals were simultaneously observed and behaviors were identified according to the “Equid Ethogram” [46]. Target behavior was not limited to the literature definition of spontaneous occurrences (SEAM), as it captured E/M that occurred in any context, including heterosexual reproductive contexts.

**Figure 1:**
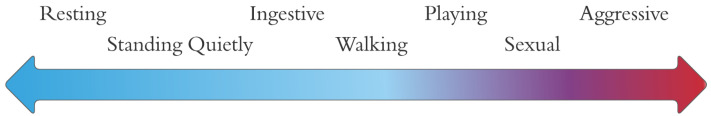
Hypothetical continuum of approximate physiological arousal levels for seven observed categories of behavior.

All observed behaviors (besides E/M) were categorized *a posteriori* into eight groups: resting, standing quietly, ingestive, walking, playing, sexual, aggressive, or other (see Table 2). These categories were chosen so that similar behaviors could be grouped (such as grazing and browsing in “ingestive”) but also account for variations in physiological arousal (for example, standing quietly and resting). Approximate levels of physiological arousal for each category are placed on a hypothetical continuum constructed for this study (see Figure 1).

The frequency of E/M was a simple count of the number of occurrences, and the duration of E/M was calculated to the nearest second. Observations were made by a live observer with a stopwatch and notebook. Bouts were also recorded with a hand-held video camera, but some of the video data were damaged in the field. In total, approximately 10 h of observations were recorded by hand.

### 2.3. Data Management and Analysis

Full descriptions of behaviors were recorded in the field. Descriptions were then labeled according to the equid ethogram [25,47] and placed into broader categories based on the equid ethogram [25,47] and study questions.

A Chi-square test of independence was used to investigate associations between category of behavior and position in behavioral sequence. This test was again used to investigate associations between changes in arousal and their timing with respect to E/M, i.e., before-after E/M, before-during E/M, and during-after E/M. Cramer’s V was used to examine the effect size of both Chi-square tests.

## 3. Results

**Figure 2:**
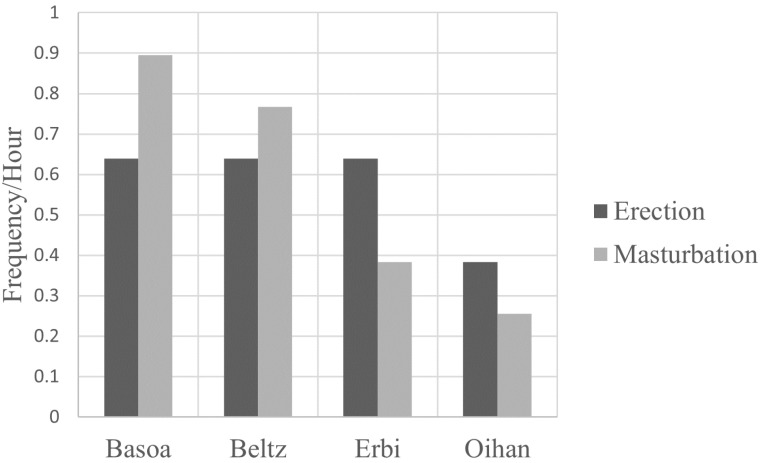
Frequency of E/M in bachelor band stallions.

In total, approximately 10.5 h of observations were collected during the study period (Table 3). During this time, 49 bouts of E/M were observed, 22 of erection only and 27 of erection with masturbation (denoted as masturbation). The range and average duration of bout are given in Table 4, and frequency of E/M of the bachelor band males is illustrated in Figure 2.

**Figure 3:**
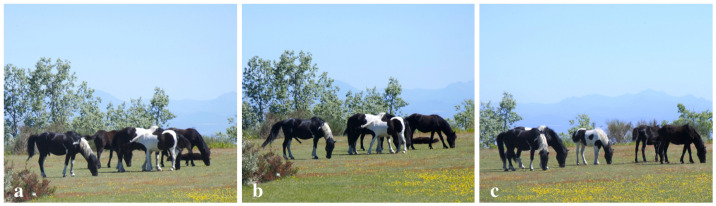
Stallion (left) performing ingestive behavior: (**a**) before E/M bout; (**b**) during E/M bout; and (**c**) after E/M bout.

**Figure 4:**
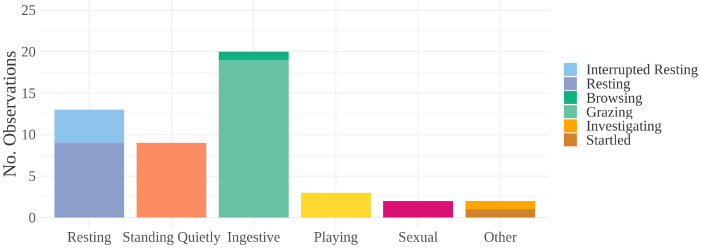
Behaviors observed immediately before an E/M bout. “Resting” includes resting and incidences of ponies that were woken from rest by the close presence of another pony. “Ingestive” includes browsing and grazing. “Other” includes one instance of startle and one instance of investigative behavior (sniffing a backpack). Arousal levels increase along the *x*-axis from left to right.

Behaviors observed directly preceding E/M include resting, standing quietly, ingestive, and playing (see Figure 3). Of these, low-arousal maintenance behaviors (resting, standing quietly, and ingestive) comprised 85.7% of the observations (42 of 49) (see Figure 4). Two observations were classified as “other”: one incident where a young bachelor band pony was sniffing a backpack before then obtaining an erection while standing quietly; another where a bachelor band pony who was resting quietly was then startled by a herd member running into him, after which he obtained an erection while standing quietly. In one incident, a stallion approached a mare, achieved an erection, and was rejected by the mare. He then moved away and stood quietly.

**Figure 5:**
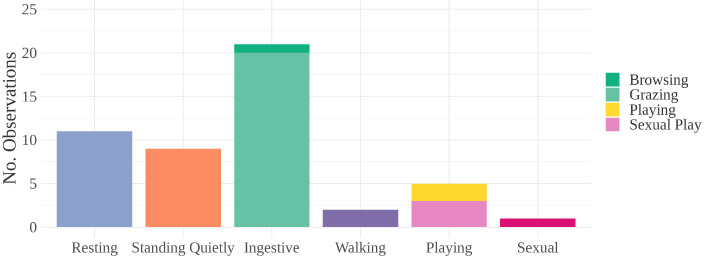
Behaviors observed concurrently with E/M bout. “Ingestive” includes browsing and grazing. “Playing” includes locomotive play (40%) and sexual play (60%). Arousal levels increase along the *x*-axis from left to right.

Behaviors observed concurrently with erection or masturbation include resting, standing quietly, ingestive, walking, playing, and sexual (see Figure 5).

In three of five play observations (two “before” and one “during”), the play is classified as sexual play (mounting behavior) between members of the bachelor bands.

**Figure 6:**
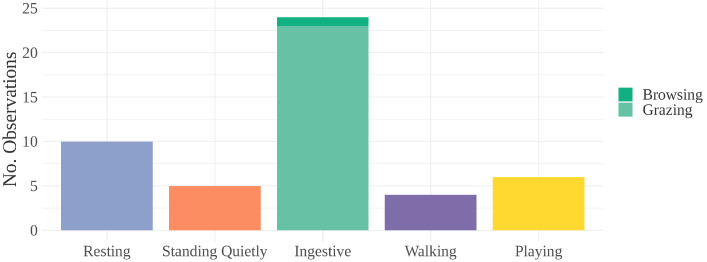
Behaviors observed immediately after E/M bout. “Ingestive” includes browsing and grazing. Arousal levels increase along the *x*-axis from left to right.

Behaviors observed after erection or masturbation include resting, standing quietly, ingestive, walking, and playing (see Figure 6). Of these, low-arousal maintenance behaviors (resting, standing quietly, ingestive) comprised 79.6% of the observation (39 of 49) (see Figure 6).

Only one aggressive incident was observed during the entire period. This incident occurred between two stallions from two different bands. One stallion and his band were in a field, and the second stallion entered the field, leaving his band behind an area of heavy brush. The interaction was brief (<3 min), and included head tossing, parallel running, sniffing and marking fecal piles, nose-to-nose contact, squealing, rearing, bucking, and striking. Following a final round of fecal pile sniffing, the second stallion retreated with his band, while the first stallion and his band remained in the field. E/M was not observed during or immediately after the aggressive behaviors. The approach of both stallions to each other was observed, and E/M was not present at this time in either stallion.

A Chi-square test of independence found no significant association between the type of behavior (ingestive, play, resting, sexual, standing quietly, walking, other) and its likelihood to be performed at a certain point in the behavioral sequences before, during, or after E/M ($\chi^{2}$ (12, 147) = 13.20, *p* = 0.35, Cramer’s V = 0.21). Additionally, a Chi-square test of independence found no significant association between the change in arousal level (increase, decrease, or no change, as defined per Figure 1) and the intervals from before-during, during-after, and before-after E/M ($\chi^{2}$ (4, N = 147) = 1.85, *p* = 0.76, Cramer’s V = 0.08).

**Figure 7:**
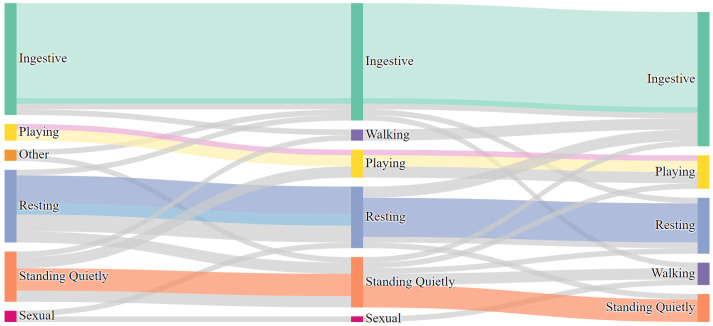
Flow of occurrences of E/M that did not change behavioral category before, during, and after E/M. The Sankey diagram, while originating as a way to show energy or materials flows through systems, can also be used to visualize a temporal sequence of events [48] or trajectory of a population through stages [49]. In this diagram, nodes represent behavioral categories Before E/M, During E/M, and After E/M (left to right). Width of node is relative to number of observations. Pathways can be traced from node to node to show flow through behavioral categories over time. Here, same-colored pathways show observations with no change in behavioral category before, during, and after E/M bout. Light blue represents one observation of interrupted rest (pony disturbed by another pony). Pink represents observations of sexual play (mounting), while remainder in play category is locomotive play.

**Figure 8:**
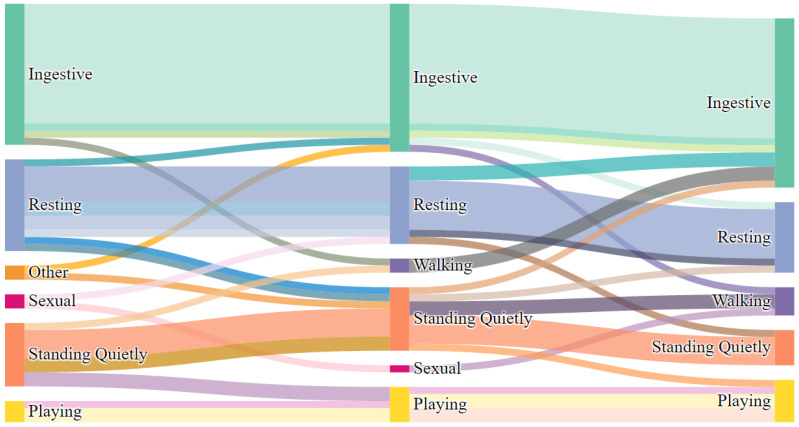
Behavioral flow of all occurrences of E/M. Nodes represent behavioral categories Before E/M, During E/M, and After E/M (left to right). Width of node is relative to number of observations. Here, all pathways can be traced between nodes to show change in behavioral category before, during, and after E/M bout. Interactive Sankey charts are available online (see Data Availability Statement).

There was no change in behavioral category before, during, and after E/M in 65.3% of observations (32 of 49; see Figure 7). There was a change in behavioral category in 34.7% of observations (17 of 49 observations; see Figure 8). In 12.2% of observations (6 of 49), behaviors flowed from categories of higher physiological arousal to lower physiological arousal (before-during-after or during-after). In 20.4% of observations (10 of 49), behaviors flowed from categories of lower physiological arousal to higher physiological arousal (before-during-after or during-after). Finally, in 2.0% of observations (1 of 49), behaviors flowed to higher physiological arousal (before to during) and then to lower physiological arousal (during to after).

## 4. Discussion and Conclusions

This study is the first to report the occurrences and types of behaviors occurring immediately before, during, and after erection or masturbation (E/M) in horses. The data suggest that E/M is a frequently occurring behavior in feral stallions. Low arousal maintenance behaviors are very likely to occur before, during, and after E/M. This study was unable to conclude that the presence of E/M predicts sexual or aggressive behavior.

The average duration and frequency of E/M observed in this study are consistent with other studies of stalled and pastured horses and donkeys. These studies found that duration of erection is approximately 2–3 min on average and occurs every 1–1.5 h, masturbation is approximately 1 minute on average and occurs 6–23 times per 24 h [1]. Comparatively, this study found the occurrence of erection in bachelor stallions to range from once every 40 min to 2 h, with an average occurrence of once per 100 min, lasting just under 2 min on average. In the present study, masturbation in bachelor stallions occurred at the same frequency as erection. While it lasted slightly longer than observed in previous studies, lasting just under 2 min (but with SD ± 1 min), the present data could be skewed as behavior was timed using a clock rather than a stopwatch.

This study also found similarities with a previous study of stalled horses regarding the behaviors co-occurring with E/M. McDonnell et al. observed 25 box-stalled horses and found that E/M co-occurred 50% of the time with ingestive behaviors (feeding and drinking) and 30% of the time with standing quietly [1]. The remainder of the co-occurrences (20%) observed in that study were concurrent with slow walking and auto-grooming. Comparatively, E/M co-occurred with ingestive behavior 42.9% of the time, and with standing quietly 18.4% of the time. Standing quietly and resting combined represented 40.8% of co-occurrences. Interestingly, despite the behavioral restrictions of stalled horses in the McDonnell studies, the frequency of E/M concurrent with ingestive and standing/resting behaviors is highly comparable to feral subjects in the present study. E/M was observed concurrent with walking 6.1% of the time and not observed concurrently with autogrooming. The present study found a larger repertoire of concurrent behaviors than that of the McDonnell studies, which can likely be attributed to the social contact and environmental space that the feral horses had to engage in sexual, play, and other behaviors. Overall, the McDonnell studies and the present study emphasize the high-frequency co-occurrence of E/M with low arousal ingestive and resting/standing behaviors in both stalled and feral horses. Furthermore, both this study and observations from McDonnell et al. found that E/M is readily truncated by environmental disturbances [1].

Although SEAM was previously studied largely in a solitary context, it was also reported to co-occur when feral or pastured males are in close proximity to other males or stalled but within visual access [24,50,51]. This study found that in 19 of 49 (39%) observations of E/M, ponies were directly touching or within one body length of another male pony. Furthermore, 10 observations (20%) occurred in four synchronous bouts. In these bouts, one or more ponies would obtain an erection and/or begin masturbating simultaneously and within one body length of another pony engaging in E/M. Within low arousal social contexts, the facial expressions and body postures did not appear qualitatively different than those performed in solitary contexts. However, two of the four bouts were observed during play sessions, where arousal was higher. While previous reports did not describe behaviors occurring before, during, or after synchronous bouts of E/M, it is perhaps not surprising that a high percentage of synchronous bouts in the present study occurred during play, as the horses most likely to have close contact with other males were also the most likely to exhibit play behavior, being both young and male. All observations of synchronous bouts occurred in the bachelor band, where social play is frequent. A study of equine play found that bouts were initiated 1.34 times per hour in young horses 18 months–3 years of age [52]. Harem stallions are more likely to engage in play than harem mares, while bachelor males show the highest amounts of play [47]. In contrast, although they may play with foals and yearlings [47], harem stallions were not observed exhibiting play behaviors in this study. As in previous studies, it is unclear if synchronous E/M is socially facilitated (synchrony is prevalent in horses [53]) or if there is a shared environmental trigger [1].

Of particular concern to horse handlers and trainers is whether or not E/M leads to sexual or aggressive behavior. This study found no evidence to support this hypothesis in feral Pottoka ponies. In 49 observations of E/M, no aggressive or sexual behaviors followed a bout of E/M. Three episodes were observed wherein sexual behavior occurred during E/M. Two were during sexual play (mounting) that then led to continued nonsexual play. Opinions differ as to whether mounting during play should be considered as agonistic [54] or a component of play [25] (a subclass of affiliative behavior [55]). While McDonnell and colleagues considered sexual play to be agonistic, their subjects were pony stallions living in crowded conditions. Furthermore, to provoke interactions such that an ethogram could be formed, the ponies lived in herds with a high amount of social instability [54]. This resulted in more frequent and more intense agonistic encounters than are reported in feral and naturally stabilized horse bands, in which aggression frequencies are low [54,56]. Social instability and crowding also possibly affected play behaviors, leading to sexual play that appeared more agonistic in the McDonnell herd than Ransom and Cade observed in feral horses in Little Book Cliffs, McCullough Peaks, and Pryor Mountain regions [25]. In the present study, sexual elements of play occurred with relatively low body and muscle tension and did not escalate into aggressive or escape behavior. Ransom and Cade consider sexual play to be a distinct behavior from spontaneous erection and masturbation (SEAM) (E/M as a comfort behavior) or reproductive E/M (observed in a heterosexual context). Furthermore, they specifically note that agonistic behaviors may be exhibited by young animals in play [25]. Accordingly, sexual play observed in this study was classified as play behavior.

This study observed two episodes of sexual behavior. One was classified as reproductive behavior, as it involved a reproductive sequence of heterosexual behavior between a stallion and a mare: the stallion showed a flehmen response after sniffing the grass (an indicator of the reproductive sequence when followed by a penis drop [25]), approached the mare with an erection, was rejected by the mare, retracted his penis, and then calmly walked out of sight. In the second observation including sexual behavior, a different stallion approached and was calmly rejected by a mare, after which he discontinued advances immediately. Of note, this stallion did not have an erection *during* his approach to the mare; it occurred after he walked away and was resting then with his eyes closed. This was not classified as reproductive behavior, as the E/M did not occur in a reproductive sequence (tending, flehmen, vocalization, rubbing, and mounting not present) and arguably not in a heterosexual context, as the stallion walked away from the mare before E/M. McDonnell and colleagues reported episodes of SEAM directly after breeding stallions covered mares, indicating that it is possible and common for SEAM to occur immediately following a reproductive opportunity, yet performed in a solitary context [1]. There were no observations of penetration or ejaculation during this study.

In the majority of observations of the present study, the arousal level of the horse did not change before, during, or after E/M, or the arousal level decreased throughout the entire sequence (77.5%). Even when arousal levels did increase, the behaviors were low arousal in general: of the total 10 observations of increased arousal, two flowed from resting to standing quietly, and three flowed from resting to walking. Additionally, no association was found between change in arousal level and the transition between behaviors for any point in the sequence (before, during, and after E/M). Accordingly, these data do not support the hypothesis that E/M behavior is associated with heightened arousal or aggression in feral horses. Although not quantified in this study, it was observed that stallions would often obtain E/M soon after waking from sleep. McDonnell and colleagues reported similar observations, as well as E/M often obtained after recovering from a startle [19]. Future studies might investigate more sensitive measures of arousal, such as parasympathetic and sympathetic nervous system activities, which mediate erection in stallions [57].

This study has a few limitations. It was conducted during a short time frame, with a relatively small number of observation hours. These hours were not balanced among harem stallions and bachelor stallions, so it is not possible to make comparisons between the two groups. Although all occurrences sampling worked well to record the behaviors of interest, it was not possible to use continuous sampling to construct full time budgets of the male ponies. Therefore, this study is unable to compare the behavioral changes associated with E/M to changes associated with other behaviors of interest, or between arousal level changes in stallions and mares. In this study, the subjects were all intact males, and previous research showed that geldings have less frequent bouts of SEAM and that bouts do not last as long [1]. However, stallions and geldings are highly similar in the manner in which SEAM is performed and the contexts in which SEAM was observed [1]. They also respond similarly to relevant pharmaceutical interventions: SEAM frequency and duration in geldings is increased with testosterone treatment, and stallion and gelding responses to imipramine hydrochloride (a drug that induces E/M) are highly similar [1]. Given that male horses are castrated (in part) to reduce aggressive and sexual behavior, the findings of this study can likely be extrapolated to geldings [22].

Based on our sample sizes and effect sizes, this study is underpowered, limiting the conclusions that can be drawn from our statistical tests. Furthermore, very few incidences of high arousal behavior, including aggression and sexual behavior, were observed. Therefore it cannot be determined if high arousal behaviors can predict or induce expressions of E/M. If the latter were to be possible, it might require either a revision of the current understanding of SEAM (a comfort behavior usually performed in a solitary context or occasionally in synchrony amongst males), or the recognition of E/M associated with aggression and sexual behavior to be distinct behavioral patterns.

Similarly, it is worth considering whether the E/M seen in handling and training contexts could be considered SEAM. While SEAM was previously defined as a spontaneous behavior, i.e., internal rather than external factors, observations of synchronous E/M in this and a previous study [51] suggest that there may be external factors contributing E/M, even if possible environmental or social triggers for the behavior are not yet understood. Likewise, E/M in a training or handling context may not be spontaneous; its triggers may be quite different from those present in male horses performing SEAM, or the social factors influencing synchronous E/M. Additionally, there were reports that E/M in training and handling contexts can differ substantially in duration, valence, and arousal. Further research of E/M in these contexts is needed.

Some equine trainers have proposed that E/M in training and handling contexts may be a displacement behavior. Displacement behaviors are performed out of context or without an apparent trigger [47,58], and are typically comfort behaviors such as eating, sniffing, scratching, rubbing the head and neck on objects, pawing, or rolling [58,59,60]. They represent a conflicted motivational state [30,47] and may function to release tension [60] or cope with stress [58]. Stabled, pastured, and feral horses regularly experience SEAM as a comfort behavior, lending plausibility to its candidacy for a displacement behavior, should the other criteria be satisfied. Further research is necessary to understand if some or all E/M during training with food is “out-of-context”, as ingestive behavior could be a stimulus for E/M, or ingestive behavior and E/M might share a common stimulus. For example, it was hypothesized that E/M is associated with return to parasympathetic dominance after a sympathetic rise [19], which could feasibly occur in a multitude of scenarios wherein an arousing environmental stimulus is followed by a quiet state [61]. Of note, the context of a human feeding a horse has no contextual parallel in the ethogram of horse–horse interactions: horses don’t feed each other; this is a novel social interaction for which no biological correspondence exists [43]. However, as McGreevy and colleagues point out, the horse–horse interaction of erection (context not specified by the authors) has an interspecies analog in one direction (horse→human) when horses are being “groomed, shod, or otherwise handled” [43]. Future studies should examine if E/M in riding, handling, and training contexts functions to cope with or reduce stress, or if there could be a separate mechanism influencing the behavior, perhaps physiological, social, or derived from some element of the human–horse interaction (such as tactile stimulation, conflicting emotional responses, underlying pain, inadvertent reinforcement, or others).

Although SEAM is thus understood as a normal and naturally occurring behavior, it does not automatically follow that all such natural behaviors should be allowed free expression without boundaries, (i.e., in contexts in which it is unsafe or intrusive to express). For example, biting and kicking, while natural behaviors [54], are unwanted both in horse–human and horse–horse interactions where the behaviors may interfere with work, sport, or cause serious injury. SEAM, however, is often observed as a low-arousal solo activity, seemingly with few safety risks. Importantly, unwanted behaviors are often indicators of pain, stress, or fear, the root cause of which should be addressed rather than the behaviors simply suppressed [62]. Perhaps more generally, this calls into question whether E/M, understood as a natural behavior, should be indicative of a problem when present in less “natural” contexts, such as within horse–human interactions. Furthermore, would such a problem(s) be hampered learning or performance, decreased human or horse safety or welfare, or is the undesirability of SEAM biased by contemporary aesthetics?

Overall, this study finds little evidence to support the hypothesis that E/M predicts aggressive or sexual behavior and little evidence that it increases emotional arousal in feral horses. Therefore, the presence of E/M is *not alone a sufficient signal* of dangerous or unwanted behavior. Rather, other behavioral signs of fear, stress, or emotional arousal, and the presentation of E/M within the behavioral sequence, might better be used to evaluate any individual horse in any unique context. It would infringe upon the welfare of stallions and geldings should they be summarily excluded from training systems that use positive reinforcement and food rewards in training due solely to the presence of E/M, and without consideration of other welfare indicators. Future research should focus on E/M within horse–human interactions.

## Figures and Tables

**Table 1:** Subjects, band membership, and age in years.

Subject	Band	Age
Basoa	Bachelor Band	2
Beltz	Bachelor Band	2
Erbi	Bachelor Band	2
Oihan	Bachelor Band	4
Indartxu	Pintxo’s Band	1
Pintxo	Pintxo’s Band	10
Ibai	Ibai’s Band	4
Gabiri	Gabiri’s Band	10

**Table 2:** Ethogram of stallion focal behaviors and categories of behaviors that occurred before and after erection and masturbation (E/M).

Ethogram Category	Description
Aggressive	Biting, kicking, striking, or chasing another horse, with
	ears pinned and muscles tense [47]
Erection	Penis fully extended and tumescent [47]
Masturbation	Bouncing or pressing of erect penis against belly [47]
Ingestive	Grazing, browsing, or drinking [47]
Play	Conspecific interactions involving locomotion and play
	fighting; including nipping, biting, pushing, bucking,
	rearing, mounting, grasping, kicking, neck wrestle, chase [25]
Walking	Four-beat locomotion [25]
Resting	Standing with eyes closed or one hind leg rested,
	lateral or sternal lying [25]
Sexual	Behavior involving a stallion and a mare that may include
	teasing, odor or pheromone detection, nudging, biting,
	mounting or attempted mounting, and copulation [25]
Standing Quietly	Standing without resting a leg, muscles relaxed, ears, head,
	and neck showing little movement *
Other	Any other behaviors; one observation of investigative
	behavior (sniffing a backpack) and one startle

* Standing Quietly, as described here, might be classified as a resting behavior in other ethograms, and the observed behavior differed from commonly used Standing Attentive [25,47]. It is used here to classify standing calmly but without attention to a specific stimulus, as in Standing Attentive.

**Table 3:** Occurrences of E/M in individual Pottoka ponies.

Subject	Status ^a^	No. Obs. E	No. Obs. M	Total Hrs Obs.	E/hour	M/hour
Gabiri	HS	1	3	>0.5 ^b^		
Ibai	HS	1	4	>1.3 ^b^		
Pintxo	HS	1	1	>0.9 ^b^		
Basoa	B	5	7	7.8 ^c^	0.6	0.9
Beltz	B	5	6	7.8 ^c^	0.6	0.8
Erbi	B	5	3	7.8 ^c^	0.6	0.4
Oihan	B	3	2	7.8 ^c^	0.4	0.3
Indartxu	Y	1	1	>0.9 ^b^		
Mean					0.6	0.6

Total observations of erection (E) and masturbation (M) for each subject during entire study. ^a^ HS = harem stallion, B = bachelor, Y = yearling. ^b^ Incomplete data regarding observation time. Therefore, no frequency is calculated for this individual. ^c^ It was not possible to record every time a pony was out of sight, however, they usually stayed in a close group where all ponies were visible.

**Table 4:** Duration of E/M in Pottoka ponies.

Behavior	No. of Obs.	Duration (Range)	Duration (Average)
Erection	22	<1–4 min (22 obs.)	1.7 min ± 0.8
Masturbation	27	<1–5 min (25 obs.)	1.8 min ± 0.9

## Data Availability

The dataset is openly available at https://doi.org/10.6084/m9.figshare.16778905.v1. Interactive charts are openly available at https://www.kaggle.com/katiegrillaert/pottoka-observations.

## Abbreviations

The following abbreviations are used in this manuscript:

E/M	Erection and Masturbation
SEAM	Spontaneous Erection and Masturbation

## References

[1] McDonnell S.M., Henry M., Bristol F. (1991). Spontaneous erection and masturbation in equids. J. Reprod. Fertil..

[2] Andersson K.E., Wagner G. (1995). Physiology of penile erection. Physiol. Rev..

[3] Wilcox S., Dusza K., Houpt K. (1991). The relationship between recumbent rest and masturbation in stallions. J. Equine Vet. Sci..

[4] McDonnell S.M. (1992). Ejaculation: Physiology and dysfunction. Vet. Clin. N. Am. Equine Pract..

[5] McDonnell S.M. (1998). Reproductive behavior of donkeys (Equus asinus). Appl. Anim. Behav. Sci..

[6] Perumal P., Vupru K., Khate K., Veeraselvam M., Verma A.K., Nahak A., Rajkhowa C. (2013). Spontaneous erection and masturbation in mithun (Bos frontalis) bulls. Int. J. Bio-Resour. Stress Manag..

[7] Jainudeen M., Hafez E. (1987). Reproductive cycles. Reproduction in Farm Animals.

[8] Marchinton R.L., Moore W.G. (1971). Auto-erotic behavior in male white-tailed deer. J. Mammal..

[9] Aronson L.R. (1949). Behavior resembling spontaneous emissions in the domestic cat. J. Comp. Physiol. Psychol..

[10] Morisaka T., Sakai M., Kogi K., Nakasuji A., Sakakibara K., Kasanuki Y., Yoshioka M. (2013). Spontaneous ejaculation in a wild Indo-Pacific bottlenose dolphin (Tursiops aduncus). PLoS ONE.

[11] Jainudeen M., Katongole C., Short R. (1972). Plasma testosterone levels in relation to musth and sexual activity in the male Asiatic elephant, Elephas maximus. Reproduction.

[12] Buechner H.K., Mackler S. (1978). Breeding behaviour in captive Indian rhinoceros. Der Zool. Gart..

[13] Michael R.P., Wilson M. (1974). Effects of castration and hormone replacement in fully adult male rhesus monkeys (Macaca mulatto). Endocrinology.

[14] Holmgren B., Urbá-Holmgren R., Trucios N., Zermeno M., Eguibar J. (1985). Association of spontaneous and dopaminergic-induced yawning and penile erections in the rat. Pharmacol. Biochem. Behav..

[15] Beach F.A. (1947). A review of physiological and psychological studies of sexual behavior in mammals. Physiol. Rev..

[16] Schiavi R.C., White D. (1976). Androgens and male sexual function: A review of human studies. J. Sex Marital. Ther..

[17] McDonnell S.M. (2000). Reproductive behavior of stallions and mares: Comparison of free-running and domestic in-hand breeding. Anim. Reprod. Sci..

[18] Mountjoy P.T. (1974). Some early attempts to modify penile erection in horse and human: An historical analysis. Psychol. Rec..

[19] McDonnell S.M., Hinze A.L. (2005). Aversive conditioning of periodic spontaneous erection adversely affects sexual behavior and semen in stallions. Anim. Reprod. Sci..

[20] Jackson J. (2014). Citizen Science—Why Do Clicker Trained Horses Drop?. http://bookendsfarm.blogspot.com/2014/04/citizen-science-why-do-clicker-trained.html.

[21] (2016). Training Not to..Ahem…Drop. https://www.horseforum.com/threads/training-not-to-ahem-drop.678938.

[22] Waring G.H. (1983). Horse Behavior. The Behavioral Traits and Adaptations of Domestic and Wild Horses, Including Ponies.

[23] Tischner M. (1982). Patterns of stallion sexual behaviour in the absence of mares. J. Reprod. Fertil..

[24] Feist J.D. (1971). Behavior of Feral Horses in the Pryor Mountain Wild Horse Range. Ph.D. Thesis.

[25] Ransom J.I., Cade B.S. (2009). Quantifying Equid Behavior—A Research Ethogram for Free-Roaming Feral Horses: U.S. Geological Survey Techniques and Methods 2-A9.

[26] Boyd L., Houpt K.A. (1994). Przewalski’s Horse: The History and Biology of An Endangered Species.

[27] Gygax L., Hillmann E. (2018). “Naturalness” and its relation to animal welfare from an ethological perspective. Agriculture.

[28] Bracke M.B., Hopster H. (2006). Assessing the importance of natural behavior for animal welfare. J. Agric. Environ. Ethics.

[29] Luescher U.A., McKeown D., Halip J. (1991). Reviewing the causes of obsessive-compulsive disorders in horses. Vet. Med..

[30] Houpt K.A., McDonnell S.M. (1993). Equine stereotypies. Comp. Cont. Educ. Pract. Vet..

[31] Marsden D. (2002). A new perspective on stereotypic behaviour problems in horses. Practice.

[32] Henderson A.J. (2021). Rising to the Occasion: Why Is My Gelding Always “Dropping”?. https://horsesport.com/magazine/behaviour/rising-occasion-why-gelding-dropping/.

[33] Weston H. (2019). Why Do Some Horses Drop Their Penis in Training?. https://connectiontraining.com/2019/06/why-do-some-horses-drop-their-penis-in-training/.

[34] Waran N., McGreevy P., Casey R. (2007). Training methods and horse welfare. The Welfare of Horses.

[35] McGreevy P.D., McLean A.N. (2009). Punishment in horse-training and the concept of ethical equitation. J. Vet. Behav..

[36] Dai F., Dalla Costa A., Bonfanti L., Caucci C., Di Martino G., Lucarelli R., Padalino B., Minero M. (2019). Positive reinforcement-based training for self-loading of meat horses reduces loading time and stress-related behavior. Front. Vet. Sci..

[37] Freymond S.B., Briefer E.F., Zollinger A., Gindrat-von Allmen Y., Wyss C., Bachmann I. (2014). Behaviour of horses in a judgment bias test associated with positive or negative reinforcement. Appl. Anim. Behav. Sci..

[38] Innes L., McBride S. (2008). Negative versus positive reinforcement: An evaluation of training strategies for rehabilitated horses. Appl. Anim. Behav. Sci..

[39] Hockenhull J., Creighton E. (2013). Training horses: Positive reinforcement, positive punishment, and ridden behavior problems. J. Vet. Behav..

[40] Lesimple C. (2020). Indicators of horse Welfare: State-of-the-Art. Animals.

[41] Mellor D.J. (2016). Moving beyond the “five freedoms” by updating the “five provisions” and introducing aligned “animal welfare aims”. Animals.

[42] Goodwin D. (1999). The importance of ethology in understanding the behaviour of the horse. Equine Vet. J..

[43] McGreevy P., Oddie C., Burton F., McLean A. (2009). The horse–human dyad: Can we align horse training and handling activities with the equid social ethogram?. Vet. J..

[44] Gonyou H.W. (1994). Why the study of animal behavior is associated with the animal welfare issue. J. Anim. Sci..

[45] Rendo F., Iriondo M., Manzano C., Estonba A. (2012). Effects of a 10-year conservation programme on the genetic diversity of the Pottoka pony–new clues regarding their origin. J. Anim. Breed. Genet..

[46] McDonnell S.M. (2003). The Equid Ethogram: A Practical Field Guide to Horse Behavior.

[47] McDonnell S.M., Poulin A. (2002). Equid play ethogram. Appl. Anim. Behav. Sci..

[48] Goulden M.C., Gronda E., Yang Y., Zhang Z., Tao J., Wang C., Duan X., Ambrose G.A., Abbott K., Miller P. (2019). CCVis: Visual analytics of student online learning behaviors using course clickstream data. Electron. Imaging.

[49] Lamer A., Laurent G., Pelayo S., El Amrani M., Chazard E., Marcilly R. (2020). Exploring patient path through Sankey diagram: A proof of concept. Digital Personalized Health and Medicine.

[50] McDonnell S., Diehl N., Garcia M., Kenney R. (1989). Gonadotropin releasing hormone (GnRH) affects precopulatory behavior in testosterone-treated geldings. Physiol. Behav..

[51] McDonnell S.M., Murray S.C. (1995). Bachelor and harem stallion behavior and endocrinology. Biol. Reprod..

[52] McCallum L., Dumbell L. (2017). Social play and its initiation in an established group of young domestic horses. Proceedings of the British Society of Animal Science.

[53] Rees L. (2017). Horses in Company.

[54] McDonnell S., Haviland J. (1995). Agonistic ethogram of the equid bachelor band. Appl. Anim. Behav. Sci..

[55] King S.R., Asa C., Pluhacek J., Houpt K., Ransom J.I. (2016). Behavior of horses, zebras, and asses. Wild Equids Ecol. Manag. Conserv..

[56] Fureix C., Bourjade M., Henry S., Sankey C., Hausberger M. (2012). Exploring aggression regulation in managed groups of horses Equus caballus. Appl. Anim. Behav. Sci..

[57] Amann R. (1981). A review of anatomy and physiology of the stallion. J. Equine Vet. Sci..

[58] Proops L., Grounds K., Smith A.V., McComb K. (2018). Animals remember previous facial expressions that specific humans have exhibited. Curr. Biol..

[59] Rees L. (1985). The Horse’s Mind.

[60] Draaisma R. (2017). Language Signs and Calming Signals of Horses: Recognition and Application.

[61] Recordati G. (2003). A thermodynamic model of the sympathetic and parasympathetic nervous systems. Auton. Neurosci..

[62] Foster R., CAAB C. Understanding and implementing principles of learning in the equine veterinary practice. Proceedings of the AAEP Annual Convention.

